# Dysregulated biosynthesis and hydrolysis of cyclic-di-adenosine monophosphate impedes sporulation and butanol and acetone production in *Clostridium beijerinckii* NCIMB 8052

**DOI:** 10.3389/fbioe.2025.1547226

**Published:** 2025-02-28

**Authors:** Marian M. Awaga-Cromwell, Santosh Kumar, Hieu M. Truong, Eric Agyeman-Duah, Christopher C. Okonkwo, Victor C. Ujor

**Affiliations:** ^1^ Department of Food Science, University of Wisconsin-Madison, Madison, WI, United States; ^2^ Biotechnology Program, College of Science, The Roux Institute, Northeastern University, Portland, ME, United States

**Keywords:** cyclic-di-adenosine monophosphate, butanol, sporulation, solventogenic clostridia, DNA integrity scanning protein A, phosphodiesterase

## Abstract

**Introduction:**

Although solventogenic *Clostridium* species (SCS) produce butanol, achieving high enough titers to warrant commercialization of biobutanol remains elusive. Thus, deepening our understanding of the intricate cellular wiring of SCS is crucial to unearthing new targets and strategies for engineering novel strains capable of producing and tolerating greater concentrations of butanol.

**Methods:**

This study investigated the potential role of cyclic-di-adenosine monophosphate (c-di-AMP) in regulating solvent biosynthesis in *C. beijerinckii* NCIMB 8052. Genes for c-di-AMP-producing and degrading enzymes [DNA integrity scanning protein A (*disA*) and phosphodiesterase (*pde*), respectively] were cloned in this organism and the recombinant strains were characterized relative to the control strain.

**Results:**

Plasmid-borne expression of disA in *C. beijerinckii* led to a 1.83-fold increase in c-di-AMP levels and near complete (∼100%) inhibition of butanol and acetone biosynthesis. Conversely, c-di-AMP concentrations in the pde-expressing strain reduced 7.54-fold relative to the control with 4.20- and 2.3-fold reductions in butanol and acetone concentrations, respectively, when compared to the control strain. Relative to the control and the *pde*-expressing strains, the *disA*-expressing strain produced 1.50- and 1.90-fold more ethanol, respectively. Enzyme activity assays show that core solvent biosynthesis enzymes are mostly inhibited *in vitro* by exogenously supplemented c-di-AMP (50 nM). Both recombinant strains of *C. beijerinckii* are impaired for sporulation, particularly the *disA*-expressing strain.

**Discussion:**

Collectively, the results show that dysregulated production and hydrolysis of c-di-AMP severely impair butanol and acetone biosynthesis in *C. beijerinckii*, suggesting broader roles of this second messenger in the regulation of solventogenesis and likely, sporulation in this organism.

## Introduction

Bio-butanol has enormous potential as a biofuel and as an industrial bulk chemical with broad applications (Agyeman-Duah et al., 2022; Qureshi and Ezeji, 2008). However, biosynthesis of butanol by solventogenic *Clostridium* species is tightly regulated, largely due to the chaotropic nature of butanol, which causes severe membrane damage with increasing concentration in the culture broth (Cray et al., 2015). Notably, butanol biosynthesis and sporulation are intricately “hardwired” in solventogenic clostridia (Long et al., 1984; Diallo et al., 2021). Consequently, the inception of butanol biosynthesis synchronizes with activation of the sporulation machinery, which acts as a cellular “shield” to protect cells against butanol toxicity by transitioning vegetative butanol-producing cells into inactive butanol-tolerant spores, with increasing butanol titer. Previously, we reported that ribonuclease P (RNase P)-mediated knockdown of the mRNA levels of the gene encoding cyclic-di-adenosine monophosphate (c-di-AMP)-producing DNA integrity scanning protein A (*disA*) in *Clostridium beijerinckii* NCIMB 8052 (hereafter, *Cbei*) increased butanol production and delayed sporulation (Ujor et al., 2021). This suggests likely implication of c-di-AMP in coordinating the interplay between sporulation and solventogenesis, at least in *Cbei*. Notably, Spo0A, the master regulator of sporulation in solventogenic clostridia has been shown to also exert a strong positive effect on butanol biosynthesis (Long et al., 1984; Diallo et al., 2021). In light of the effect of *disA* knockdown on butanol production and sporulation, the role of Spo0A in solventogenesis and sporulation, and the established role of c-di-AMP in regulating sporulation in *Bacillus subtilis* (Oppenheimer-Shaanan et al., 2011), it is plausible that c-di-AMP might directly or indirectly influence the interplay between sporulation and solventogenesis in SCS.

DisA is a diadenylate cyclase that produces the bacterial second messenger, c-di-AMP (Bejerano-Sagie et al., 2017; Teh et al., 2019). C-di-AMP exerts far-reaching effects on the overall biology of producer organisms; playing significant roles in sporulation, DNA repair, cell wall synthesis/cross linking, response to osmotic stress and maintenance of cell envelope integrity; virulence, regulation of gene expression, and control of cellular fitness (Bejerano-Sagie et al., 2017; Oppenheimer-Shaanan et al., 2011; Commichau et al., 2015; Rørvik et al., 2021). Among these roles of c-di-AMP, its involvement in sporulation and maintenance of cell envelope integrity might interface with the regulation of butanol biosynthesis and tolerance. Given the chaotropic nature of butanol, hence, its membrane damaging effect (Cray et al., 2015) and the ability of c-di-AMP to regulate response to osmotic stress/damage, it is possible that c-di-AMP might serve as a nimble cellular “switch” that links response to butanol biosynthesis, butanol toxicity, and sporulation in *Cbei*.

In this study, we sought to further investigate the exact roles that c-di-AMP might play in butanol production and tolerance, and sporulation in *Cbei*. Cloning and overexpressing the native *disA* and a c-di-AMP-specific (hydrolyzing) phosphodiesterase gene (*pde*; encoding a protein homologous to DHH/DHHA1 phosphodiesterase in *B. subtilis*) in *Cbei* showed that dysregulated biosynthesis and hydrolysis of c-di-AMP severely impairs or completely inhibits butanol production in this organism. *In vitro*, c-di-AMP exhibits dose dependent inhibition of the activities of some butanol and acetone biosynthesis enzymes. Herein, we present different lines of evidence that further link intracellular c-di-AMP levels to the regulation of solvent production and sporulation in *Cbei*.

## Materials and methods

### Bacterial strains and culture media

*Escherichia coli* DH5α was procured from New England Biolabs (Ipswich, MA, United States). *Escherichia coli* DH5α was propagated in Luria-Bertani (LB) broth. *Cbei* was acquired from the American Type Culture Collection (Manassas, VA, United States) and maintained in the laboratory as a spore suspension in sterile distilled water, according to a previously reported protocol (Agyeman-Duah et al., 2022).

### Engineering c-di-AMP over-producing and hydrolyzing strains of *C. beijerinckii*

The primers used to clone *Cbei*_*disA* (::*disA*) and *Cbei*_*pde* (::*pde*) are presented in Supplementary Table S1 (Supplementary Material). The genes *disA* (Cbei_0127) and *pde* (Cbei_5082) were amplified from the genomic DNA of *Cbei* extracted according to the method described by Agyeman-Duah et al. (2022). Two-step amplification was used for each gene wherein, the first sets of primers (Cbei_0127_Fv1 + Cbei_0127_Rv1 and Cbei_5082_Fv1 + Cbei_5082_Rv1; Supplementary Table S1) were used solely for gene amplification and the second sets (Cbei_0127_Fv2 + Cbei_0127_Rv2 and Cbei_5082_Fv2 + Cbei_5082_Rv2) were used to incorporate *ApaI* and *XhoI* restriction sites into the amplicons. Amplification was conducted with Phusion Taq DNA polymerase (New England Biolabs, Ipswich, MA, United States) according to the following reaction conditions: 98°C for 2 min; 15 cycles of 98°C for 20 s, 50°C for 15 s, and 72°C for 2 min followed by 21 cycles of 98°C for 20 s, 63°C for 15 s and 72°C for 9 min and a final hold at 4°C. Both *disA* and *pde* were cloned into the *Clostridium*-*E. coli* shuttle vector pWUR459 (Siemerink et al., 2011) following digestion with *ApaI* and *XhoI* (New England Biolabs, Ipswich, MA, United States). The plasmid constructs were chemically transformed into *E. coli* Dh5α (Agyeman-Duah et al., 2024) and colonies were selected on LB agar plates supplemented with ampicillin (100 μg/mL). Positive transformants were confirmed by colony PCR and sequencing of PCR amplicon and plasmid constructs using gene-specific and M13 forward and reverse primers (Eurofins Genomics, Louisville, KY, United States). Subsequently, the plasmid constructs were electro-transformed into *Cbei* following a previous protocol (Agyeman-Duah et al., 2024). The transformants were grown on tryptone-glucose-yeast extract (TGY) agar (0.5%, *w*/*v*). Positive colonies were selected, grown in TGY medium and then stored as glycerol stocks [30% glycerol (v/v)] at −80°C for future experiments. Additionally, the recombinant strains were grown to sporulation in P2 fermentation medium (100 mL) containing glucose (60 g/L), yeast extract (1 g/L), and 1 mL each of pre-filter-sterilized mineral, buffer and vitamin stocks in loosely capped 250-mL Pyrex culture bottles. The buffer stock comprised of (in g/L): K_2_HPO_4_ (50), KH_2_PO_4_ (50) and ammonium acetate (220). The mineral stock contained (in g/L): MgSO_4_.7H_2_O (20) MnSO_4_·H_2_O (1), FeSO_4_.7H_2_O (1), and NaCl (1), while the vitamin stock contained (in g/L): *p*-amino-benzoic acid (0.1), thiamine (0.1), and biotin (0.001). The spores were harvested after 2 weeks, washed five times in sterile distilled water and stored in sterile distilled water at 4°C.

### Fermentation

Fermentation of glucose and arabinose by *Cbei*_p459 (empty plasmid control), *Cbei*_*disA* and *Cbei*_*pde* was conducted in P2 medium described above. Spores (200 μL) of *Cbei*_*disA*, *Cbei*_*pde*, and *Cbei*_p459 were inoculated into 50 mL of TGY broth and grown overnight as precultures. Afterwards, the precultures (6% *v*/*v*) were transferred into P2 medium containing 60 g/L glucose or arabinose. Arabinose was used as substrate because we previously observed improved butanol production in *Cbei* following RNase P-mediated knockdown of *disA* (Ujor et al., 2021). The cultures were supplemented with 25 µg of erythromycin and samples were taken every 12 h and analyzed for acetone-butanol-ethanol (ABE), acetic and butyric acids concentrations; optical density, and pH. Except where stated otherwise, all cultures were grown and analyzed in triplicate. All cultures were grown at 35°C ± 1°C in an anaerobic chamber (Coy Laboratory Products Inc., Grass Lake, MI, United States) with a modified atmosphere of 82% N_2_, 15% CO_2_, and 3% H_2_.

### Analytical procedures

The concentrations of ABE, acetic and butyric acids were quantified using a Shimadzu GC-2010 Plus gas chromatography (Shimadzu, Columbia, MD, United States) equipped with a flame ionization detector (FID) and a Shimadzu SH-PolarWax crossbond carbowax polyethylene glycol column [30 m (length), 0.25 mm (internal diameter), and 0.25 μm (film thickness)] according to a previous method (Agyeman-Duah et al., 2022). Culture pH was monitored using an Orion Star A214 pH meter (ThermoFisher Scientific, Waltham, MA, United States) and cell growth was measured as optical density (OD_600_ nm) with an Evolution 260 Bio UV/Visible spectrophotometer (ThermoFisher Scientific, Waltham, MA, United States).

### Quantitative real time polymerase chain reaction (qRT-PCR)

To isolate RNA, *Cbei*_*disA Cbei*_*pde*, and *Cbei*_p459 were grown in P2 medium as described above. RNA was isolated from cell samples collected at 24 h and 36 h. RNA isolation was carried out using TRIzol™ Reagent (ThermoFisher Scientific, Waltham, MA, United States) according to an earlier described method (Kumar et al., 2024). Complementary DNA (cDNA) was synthesized and used as a template for qRT-PCR using gene-specific primers as described by Kumar et al. (2024). The expression profiles of 20 genes were studied by qRT-PCR (Supplementary Table S1). Relative gene expression was quantified by normalizing the target genes to *rpoD* and *16SRNA* housekeeping genes of *Cbei* using the 2^−ΔΔCt^ method. Results are represented as the means ± standard deviation of three technical replicates.

### Quantification of intracellular concentrations of c-di-AMP

Intracellular levels of c-di-AMP were quantified according to the method described by Huynh et al. (2015). *Cbei*_*disA*, *Cbei*_*pde*, and *Cbei*_p459 were grown in P2 medium as described earlier (under fermentation). After 24 h, 2 mL samples were drawn and centrifuged at 16,000 × g for 10 min (Sorvall Legend Micro 21R, ThermoFisher Scientific, Waltham, MA, United States). The cell pellets were re-suspended in 50 µL of heavy isotope (C and N) labeled c-di-AMP and 500 µL of methanol. Cells were lysed on ice in the sonicator at 80% amplitude for 10 pulses (1 s on/1 s off for 20 s). After lysis, the mixture was centrifuged as described above and the supernatant was transferred into a new centrifuge tube. Subsequently, 500 µL of methanol was added to the pellet portion, vortexed, and centrifuged as described above. The resulting supernatant was added to the initial supernatant. The pooled supernatants were concentrated in an Eppendorf Vacufuge Plus 5305 (Eppendorf, Enfield, CT, United States) for 2 h with the operation and application mode set to vacuum alcohol. Afterwards, the pellets were rehydrated in 50 µL of sterile double distilled water. The extracted c-di-AMP was quantified by LC/MS (Sciex 5500 QTRAP triple quadrupole/ion trap mass spectrometer connected to an Agilent 1100 Nano flow HPLC system). Chromatographic separation was achieved on a Synergi 4u Hydro-RP 80A column (50 × 2 mm, 4 μM particle size; Phenomenex) with a mobile phase consisting of 10 mM formic acid in water (Solvent A) and 10 mM formic acid in methanol (Solvent B) as previously described by Huynh et al. (2015). Intracellular amounts of c-di-AMP levels are represented as average values of three biological replicates in µg/g cell dry weight.

### Enzyme activity assays

*Cbei*_*disA*, *Cbei*_*pde*, and *Cbei*_p459 were grown on glucose (P2 medium) as earlier described and butanol dehydrogenase (BDH), butyraldehyde dehydrogenase (BDDH), acetoacetate decarboxylase (ADC) and coenzyme A transferase (CoAT) activity assays were performed on crude cell extracts according to the methods of Agyeman-Duah et al. (2022), with slight modifications. Cells were harvested after 24 h, washed, pelleted by centrifugation as described under c-di-AMP quantification, and then lysed in 1 mL of lysis buffer (10 mg/mL lysozyme, 10 mM phosphate buffer and 1 mM phenylmethylsulfonyl fluoride) for 1 h at 37 °C. BDH activity was quantified with butyraldehyde as substrate by measuring the decrease in absorbance (due to NADPH consumption) at 340 nm (0.1 mM NADPH was used). BDDH activity was measured with butyryl coenzyme A by monitoring the decrease in absorbance at 340 nm (due to NADH consumption). ADC activity was quantified by measuring the decrease in the absorbance of acetoacetate at 270 nm in a reaction mixture containing 50 mM potassium phosphate (pH 5.9), 300 mM lithium acetoacetate, and 50 µL of the crude cell extract (Fridovich, 1972). To assess possible effect of c-di-AMP on enzyme activities, the assay reaction mixtures for BDH, BDDH, ADC, and CoAT were supplemented with 5 nM or 50 nM sodium salt of c-di-AMP (Millipore-Sigma, Burlington, MA, United States). Assay mixtures without the respective substrates, cell lysate, and c-di-AMP were used as the negative controls. The molar extinction coefficients used to calculate enzyme activities include 6220 M^-1^cm^-1^ for NADH and NADPH at 340 nm, 55 M^-1^cm^-1^ for acetoacetate at 270 nm, and 7800 M-1cm-1 for acetoacetyl-CoA at 310 nm. Protein concentrations of the lysates were measured and enzyme activities were expressed in units of activity per mg protein.

### Butanol tolerance assay

To assess the butanol tolerance of each strain, cultures were grown as described earlier (under fermentation). After 24 h, 12 g/L of butanol was added to each culture. Sterile distilled water was added to the control culture. Eight hours after butanol addition, triplicate samples were drawn and vegetative cell mass was measured as a function of optical density using Evolution 260 Bio UV/Visible spectrophotometer (ThermoFisher Scientific, Waltham, MA, United States). Samples were taken from the top quarter part of the culture bottles to avoid taking up spores, which settle at the bottom of the culture. To determine if classic butanol-mediated membrane damage mimics KCl-induced osmotic shock, the cultures were challenged with 12 g/L butanol and 25 mM and 50 mM KCl. The cultures were challenged at 24 h. Four hours after the challenge, 10 mL samples were taken and processed for scanning electron microscope as described below.

### Phase contrast microscopy

*Cbei*_*disA*, *Cbei*_*pde*, and *Cbei*_p459 were grown in triplicate on glucose as described under fermentation and 1 mL samples were taken after 24 h for phase contrast microscopy (Nikon Eclipse Ti series microscope, Nikon, Melville, NY, United States). Phase contrast microscopy was carried out according to our previously reported method (Kumar et al., 2024). All samples were viewed and captured at ×100 magnification. The number of endospore-bearing cells relative to total vegetative cells were enumerated in triplicate to determine percentage spore formation for each strain of *Cbei*.

### Scanning electron microscopy (SEM)

Cultures of *Cbei*_*disA*, *Cbei*_*pde*, and *Cbei*_p459 were grown as described above. After 24 h, 2 mL samples were taken and prepared for SEM according to the method described by Pilavtepe-Çelik et al. (2008) with slight modifications. Cell pellets were collected by centrifuging the samples at 600 × g for 5 min at room temperature. Cell pellets were washed twice with phosphate buffered saline (pH = 7.4) and centrifuged as above. Subsequently, cells were washed in 1 mL of 0.2 M sodium cacodylate buffer (pH = 7.4) and centrifuged twice as above. The cells were re-suspended in 1 mL of fixative (2% paraformaldehyde, 2.5% glutaraldehyde in 0.1M sodium cacodylate Buffer, pH 7.4) for 2 h at room temperature, and then stored overnight at 4 °C. The fixed samples were washed twice with 0.2 M sodium cacodylate buffer and centrifuged as above for 10 min. The fixed cells were then dehydrated in an ethanol series (25%, 50%, 70%, 95% and 100%) in a rotary shaker at 200 rpm. Each dehydration step lasted for 15 min, and ethanol was removed by centrifuging for 10 min at 2,500 rpm. The last dehydration step in 100% ethanol was performed three times with fresh ethanol. Afterwards, the samples were treated three times with 100% hexamethyldisilazane (HMDS) for 15 min/cycle. After the third HMDS treatment, 5 μL of each sample was pipetted onto a silicon chip and air-dried for 30 min in a humid chamber. After drying, the silicon chips were secured onto SEM stubs using 25 mm double-sided carbon adhesive tabs. The prepared SEM stubs were placed into the sputter coater (Quorum, Model: Q150T S plus) and the chamber was evacuated to create a vacuum. Consequently, the samples were sputter coated with 3 mm of platinum while monitoring the coating process to ensure a uniform coating. The samples were carefully transferred into the SEM chamber (Hitachi, Model: SU5000) and the SEM parameters were optimized for imaging. The accelerating voltage was set to 5–15 kV and the working distance was set to 5–10 mm. Samples were imaged and the high-resolution version of the images was captured while adjusting the focus, magnification, and contrast as needed to obtain clear and detailed images.

### Bioinformatics

To identify putative c-di-AMP-binding proteins in *Cbei*, the conserved c-di-AMP binding motifs SXSX_20_-FTAXY and the RCK_C domain (Moscoso et al., 2015; Blötz et al., 2017) were blasted against the *Cbei* proteome using the National Center for Biotechnology Information Protein BLAST tool.

### Statistical analysis

Analysis of variance (ANOVA) test was conducted to compare the results of the treatment and control groups using RStudio (version 2024.09.0 + 375; Posit, PBC, MA, United States). The c-di-AMP concentrations, butanol tolerance, and enzyme activities of the three strains of *Cbei* studied were analyzed at 95% confidence interval and treatments with a p ≤ 0.05 were considered to show evidential difference. Also, Tukey’s Honest Significant Difference (HSD) test was employed to identify the treatments with evidently different means. All treatments were analyzed in triplicates (*n* = 3).

## Results

### Overexpressing *disA* and *pde* in *Cbei* leads to over- and under-production of c-di-AMP

**FIGURE 1:**
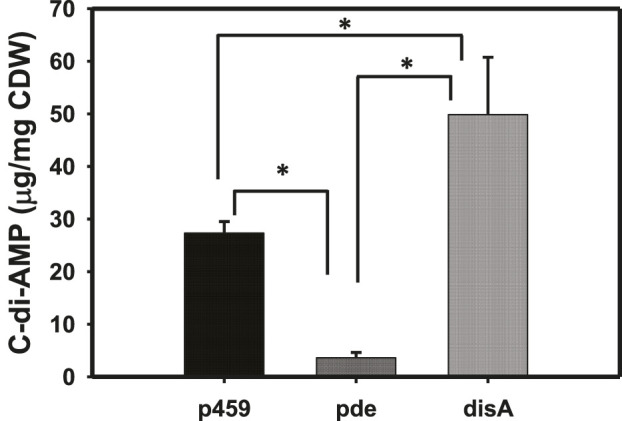
C-di-AMP profiles in *Cbei*_p459, *Cbei*_*pde*, and *Cbei*_*disA*. *P*-values were calculated using c-di-AMP concentrations from three biological replicate cultures. p459 = *Cbei*_p459; pde = *Cbei*_*pde*; disA = *Cbei*_*disA*. Asterisks (*) denote statistical significance (*p* < 0.05) relative to the compared strain.

As expected, overexpressing *disA* and *pde* in *Cbei* resulted in significantly higher and lower intracellular concentrations of c-di-AMP, respectively (Figure 1). The levels of c-di-AMP were 1.83- and 4.20-fold higher and lower in *Cbei*_*disA* and *Cbei*_*pde*, respectively, relative to *Cbei*_p459 (p < 0.05). Compared to *Cbei*_*pde*, *Cbei*_*disA* contained 13.80-fold more c-di-AMP (p < 0.05).

### Over- and under-production of c-di-AMP impairs growth and sporulation and inhibits acetone and butanol but not ethanol biosynthesis

**FIGURE 2:**
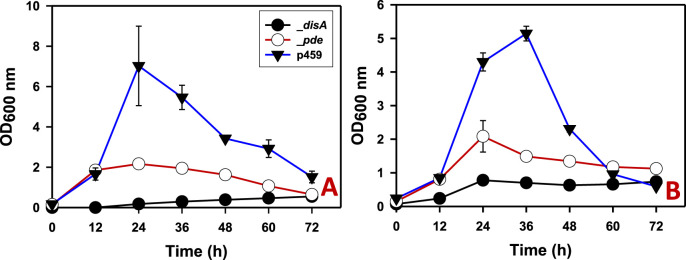
The growth profiles of *Cbei*_*disA*, _*pde*, and _p459 on glucose and arabinose. **(A)** Glucose. **(B)** Arabinose. Dysregulated expression of *disA* and *pde* impaired growth in *Cbei*_*disA* and *Cbei*_*pde* relative to *Cbei*_p459. Data is presented as the mean of three biological replicates (n = 3). Error bars represent standard deviation. _*disA* = *Cbei*_*disA*; _*pde* = *Cbei*_*pde*; p459 = *Cbei*_p459.

On both glucose and arabinose, overexpressing *disA* and *pde* significantly impaired growth in *Cbei*. Growth impairment was more pronounced in cultures of *Cbei*_*disA* which exhibited 6.60- and 12.80-fold reduced maximum optical densities on arabinose and glucose (p < 0.05), respectively, relative to *Cbei*_p459 (Figure 2). On the other hand, the maximum optical densities reached by *Cbei*_*pde* were 2.50- and 3.30-fold less than those recorded for *Cbei*_p459 on arabinose and glucose respectively (p < 0.05).

**FIGURE 3:**
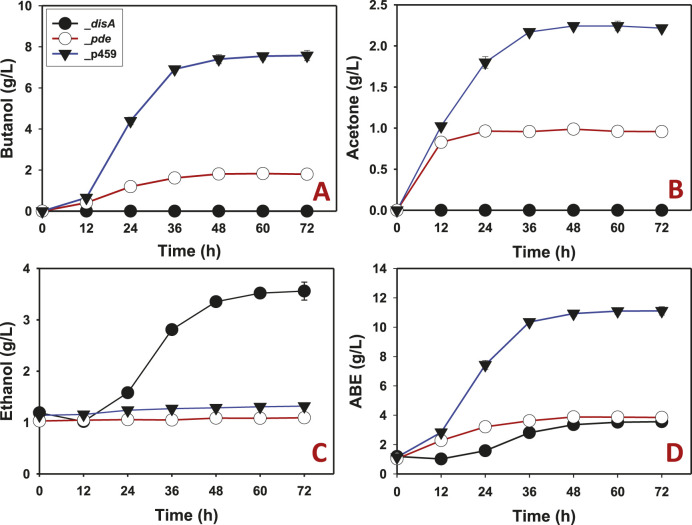
Solvent profiles of *Cbei*_p459, *Cbei*_*pde*, and *Cbei*_*disA* grown on glucose. **(A)** Butanol. **(B)** Acetone. **(C)** Ethanol. **(D)** ABE. Dysregulated expression of *disA* inhibited butanol and acetone biosynthesis. Dysregulated expression of *pde* impaired butanol and acetone production. Data is presented as the mean of three biological replicates (n = 3). Error bars represent standard deviation.

Butanol and acetone biosyntheses were completely inhibited in *Cbei*_*disA* grown on glucose (Figures 3A, B). Comparatively, *Cbei*_p459 and *Cbei*_*pde* produced maximum butanol and acetone concentrations of 7.58 and 2.43 g/L and 1.83 and 1.00 g/L, respectively. Remarkably, *Cbei*_*disA* produced up to 2.73-fold more ethanol than *Cbei*_p459 and 3.30-fold more than *Cbei*_*pde* (p < 0.05; Figure 3C). Overall, *Cbei*_*disA* and *Cbei*_*pde* produced similar maximum concentrations of ABE (total solvents = acetone + butanol + ethanol; 3.60 and 3.9 g/L, respectively). These were at least 2.90-fold less than the maximum concentration detected in cultures of *Cbei*_p459 (p < 0.05; Figure 3D).

**FIGURE 4:**
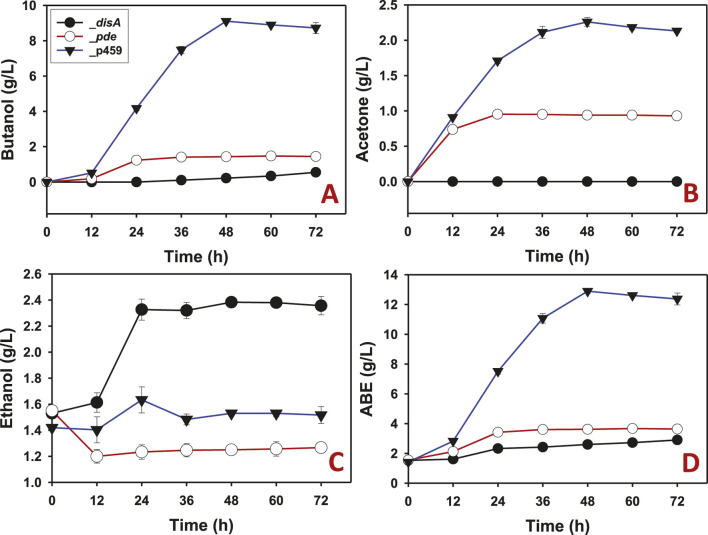
The solvent profiles of *Cbei*_p459, *Cbei*_*pde*, and *Cbei*_*disA* grown on arabinose. **(A)** Butanol. **(B)** Acetone. **(C)** Ethanol. **(D)** ABE. Dysregulated expression of *disA* inhibited butanol and acetone biosynthesis. Dysregulated expression of *pde* impaired butanol and acetone production. Data is presented as the mean of three biological replicates (n = 3). Error bars represent standard deviation.

When arabinose was used as substrate, similar solvent profiles were observed for *Cbei*_*disA*, *Cbei*_*pde*, and *Cbei*_p459 compared to glucose. As with glucose, butanol production was largely inhibited in arabinose-grown *Cbei*_*disA*, although a delayed marginal butanol production was observed with this strain. Whereas butanol was detected at 12 h in cultures of *Cbei*_*pde* and *Cbei*_p459, butanol was not detected in cultures of *Cbei*_*disA* until 36 h. A total of 0.55 g/L butanol was produced by *Cbei*_*disA*, which was 2.62- and 16.60-fold less than the maximum butanol titers produced by *Cbei*_*pde* and *Cbei*_p459, respectively (p < 0.05; Figure 4A). As observed with glucose, acetone biosynthesis was completely inhibited in *Cbei*_*disA* grown on arabinose whereas *Cbei*_p459 and *Cbei*_*pde* produced 2.26 and 0.95 g/L acetone, respectively (Figure 4B). With arabinose as substrate, *Cbei*_*disA* produced 1.50-fold more ethanol than *Cbei*_p459 and 1.90-fold more than *Cbei*_*pde* (p < 0.05; Figure 4C). *Cbei*_*disA* and *Cbei*_*pde* produced similar ABE titers (2.90 and 3.67 g/L, respectively), which were at least 3.51-fold less than that produced by *Cbei*_p459 (p < 0.05; Figure 4D). Apparently, inhibition of butanol production in *Cbei*_*disA* elicited greater ethanol production, which accounts for the similarities in maximum ABE concentrations between *Cbei*_*disA* and *Cbei*_*pde* (Figures 3D, 4D).

### C-di-AMP inhibits solvent biosynthesis enzymes in *Cbei in vitro*.

**FIGURE 5:**
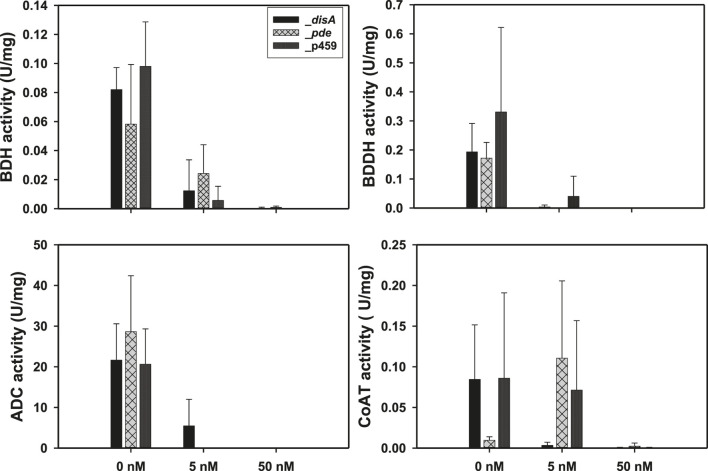
Exogenous supplementation of 50 nM c-di-AMP inhibited the activities of butanol dehydrogenase (BDH), butyraldehyde dehydrogenase (BDDH), acetoacetate decarboxylase (ADC), and coenzyme A transferase (CoAT). Activities are expressed in units per mg protein (U/mg). Data is presented as the mean of three biological replicates (n = 3). Error bars represent standard deviation.

Exogenous supplementation of the enzyme assay mixtures with c-di-AMP resulted in a concentration dependent inhibition of BDH, BDDH, and ADC activities (Figure 5). With 5 nM c-di-AMP, at least 2.50-, 50.80-, and 4.00-fold reductions in activity were observed for BDH, BDDH, and ADC, respectively (p < 0.05). When the concentration of c-di-AMP was increased to 50 nM, ∼100% inhibition of activity was observed for BDH, BDDH, and ADC (*p* < 0.05). No significant inhibition in activity was observed for CoAT with 5 nM c-di-AMP (Figure 5). However, when c-di-AMP concentration was increased to 50 nM, CoAT activity was almost completely diminished.

### Over- and under-production of c-di-AMP altered gene expression patterns

Compared to *Cbei*_p459, coenzyme A transferase genes (*ctfAB*) that encode enzymes involved in the reabsorption of acetic and butyric acids were significantly upregulated in *Cbei*_*disA* and *Cbei*_*pde* at 24 h (Supplementary Figure S1; Table 1; *p* < 0.05). At 36 h, whereas these genes remained upregulated in *Cbei*_*disA* relative to *Cbei*_p459, they were significantly downregulated in *Cbei*_*pde* (p < 0.05; Supplementary Figure S1; Table 1). *Cbei* has five butanol dehydrogenase genes (*adhE1*, *adhE2*, *bdhA*, *bdhB1* and *bdhB2*) that code for enzymes annotated to catalyze the final step in butanol biosynthesis (Zhao et al., 2020). These genes exhibited varying mRNA abundances in *Cbei*_*disA* and *Cbei*_*pde* relative to *Cbei*_p459. Specifically, at 24 h *adhE1*, *adhE2*, *bdhA*, *bdhB1*, and *bdhB2* were either unchanged or downregulated in *Cbei*_*disA* compared to *Cbei*_p459 (Table 1; Supplementary Figure S2; *p* < 0.05). However, at 36 h, with the exception of *bdhB2*, which was downregulated, all the butanol dehydrogenase genes studied were significantly upregulated in *Cbei*_*disA* relative to *Cbei*_p459 (p < 0.05). Interestingly, all the butanol dehydrogenase genes studied were either unchanged or downregulated in *Cbei*_*pde* relative to *Cbei*_p459 (Table 1; Supplementary Figure S2; *p* < 0.05). Acetoacetate decarboxylase gene (*adc*), of which the protein product catalyzes the final step in acetone biosynthesis was upregulated at both time points in both recombinant strains of *Cbei* except at 24 h in *Cbei*_*pde* (*p* < 0.05). Similarly, pyruvate carboxylase gene (*pyc*; Cbei_4960) was upregulated at both time points in *Cbei*_*disA*. On the other hand, *pyc* was upregulated at 24 h and downregulated at 36 h in *Cbei*_*pde* when compared to *Cbei*_p459.

**TABLE 1:** mRNA fold changes for select genes in *Cbei*_*disA* and *Cbei*_*pde* relative to *Cbei*_p459.

Gene name	ORF	mRNA fold change			
		*Cbei*_*disA* vs. *Cbei*_p459		*Cbei*_*pde* vs. *Cbei*_p459	
		24 h	36 h	24 h	36 h
*cftA*	Cbei_2654	2.90 ± 0.15	2.35 ± 0.24	1.45 ± 0.06	−4.55 ± 0.27
*cftA*	Cbei_3833	2.06 ± 0.21	1.56 ± 0.016	4.05 ± 0.06	−2.04 ± 0.12
*ctfB*	Cbei_2653	3.21 ± 0.23	3.27 ± 0.22	3.10 ± 0.04	−1.96 ± 0.31
*ctfB*	Cbei_3834	2.16 ± 0.06	3.23 ± 0.15	3.68 ± 0.33	−1.17 ± 0.11
*adhE1*	Cbei_0305	1.04 ± 0.06	1.89 ± 0.04	−2.85 ± 0.54	−31.01 ± 8.49
*adhE2*	Cbei_4053	−1.74 ± 0.26	2.03 ± 0.23	−0.35 ± 1.95	−85.39 ± 15.85
*bdhA*	Cbei_2421	−1.21 ± 0.09	2.64 ± 0.08	−3.98 ± 0.50	−1.98 ± 0.16
*bdhB1*	Cbei_2181	−1.09 ± 0.01	2.26 ± 0.17	1.10 ± 0.03	1.30 ± 0.12
*bdhB2*	Cbei_1722	−1.97 ± 0.51	−0.70 ± 1.61	−1.35 ± 0.08	1.15 ± 0.09
*adc*	Cbei_3835	1.33 ± 0.05	5.60 ± 0.14	−1.36 ± 0.02	2.62 ± 0.17
-	Cbei_4960	1.44 ± 0.38	3.25 ± 0.67	3.12 ± 0.31	−0.86 ± 1.78
*spo0A*	Cbei_1712	2.82 ± 0.37	1.25 ± 0.06	−2.28 ± 0.09	1.34 ± 0.27
*sigE*	Cbei_1120	2.88 ± 0.31	1.82 ± 0.11	1.47 ± 0.21	2.37 ± 0.31
*σ* ^*70*^	Cbei_4990	6.47 ± 0.36	2.01 ± 0.03	1.58 ± 0.23	−13.16 ± 3.72
-	Cbei_0017	−37.09 ± 8.16	14.04 ± 1.43	1.40 ± 0.19	18.70 ± 0.62
-	Cbei_3078	−0.35 ± 1.39	2.67 ± 0.11	−0.34 ± 1.18	−2.60 ± 0.86
*cdaA*	Cbei_0200	−0.34 ± 1.23	1.49 ± 0.15	−1.46 ± 5.21	3.52 ± 0.14
*disA*	Cbei_0127	1.11 ± 0.08	1.24 ± 0.13	1.69 ± 0.18	1.36 ± 0.07
*gdpP*	Cbei_1538	−3.52 ± 1.06	−0.47 ± 1.37	−5.98 ± 0.57	−3.01 ± 0.67
*pde*	Cbei_5082	9.54 ± 1.38	8.90 ± 0.61	206.83 ± 9.76	317.87 ± 4.74

ORF, Open reading frame; *n* = 3

With the exception of Cbei_3078 (which codes for PAS/PAC sensor hybrid histidine kinase) that was mostly downregulated, the genes involved in sporulation, transcription, and signal transduction that were studied (*spo0A*, *sigE*, *σ* ^*70*^, and Cbei_0017 - histidine kinase) were mostly upregulated in *Cbei*_*disA* and *Cbei*_*pde* when compared to *Cbei*_p459 at 24 and 36 h (Table 1; *p* < 0.05). As expected, *disA* exhibited higher mRNA abundances in *Cbei*_*disA* and *Cbei*_*pde* at 24 and 36 h (*p* < 0.05). Interestingly, *cdaA*, which codes for a protein with c-di-AMP synthetase/DisA_N domain was downregulated in *Cbei*_*disA* and *Cbei*_*pde* at 24 h, whereas the mRNA increased in abundance at 36 h (particularly in *Cbei*_*pde*), when compared to *Cbei*_p459. Similarly, the abundance of *pde* mRNA was significantly greater in *Cbei*_*disA* and *Cbei*_*pde* at 24 and 36 h, relative to the empty plasmid control strain. Conversely, *gdpP* (c-di-AMP-specific phosphodiesterase) was downregulated in *Cbei*_*disA* and *Cbei*_*pde* at 24 and 36 h (*p* < 0.05).

### Plasmid-borne expression of *disA* or *pde* caused delayed sporulation in *Cbei*

**FIGURE 6:**
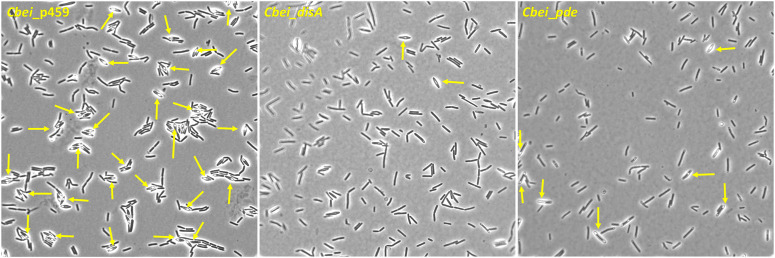
Sporulation is delayed in *Cbei*_*disA* and *Cbei*_*pde* relative *Cbei*_p459. Arrows show endospores. Samples were viewed with ×100 magnification.

**FIGURE 7:**
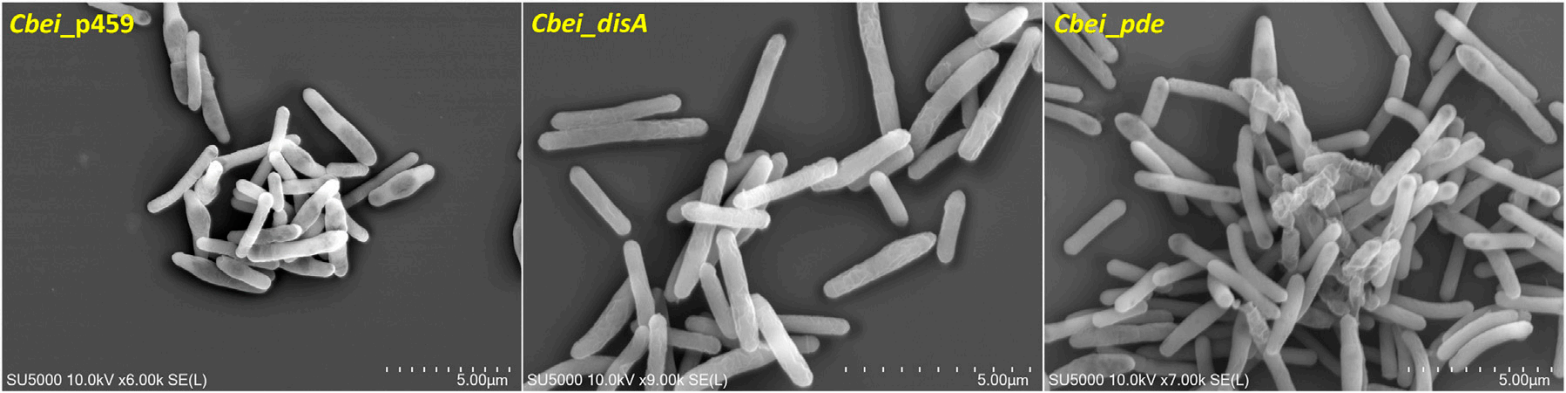
Delayed sporulation in *Cbei*_*disA* and *Cbei*_*pde* relative *Cbei*_p459 and rupturing in *Cbei*_*pde*.

Butanol biosynthesis and sporulation occur simultaneously in solventogenic *Clostridium* species, with the rate of sporulation increasing with increasing butanol concentration in the culture (Long et al., 1984; Diallo et al., 2021). Previously, using a spore germination count approach we showed that knockdown of *disA* elicited up to 7.4-fold reduction in the rate of percentage spore formation relative to wildtype *Cbei*. In this study, we used microscopy to qualitatively confirm delayed sporulation in *Cbei* because of high and/or low c-di-AMP production. Phase contrast microscopy revealed extensive formation of endospores (58% spore formation) in *Cbei*_p459 at 24 h (Figure 6). Conversely, endospores were sparsely detected in cultures of *Cbei*_*disA* (1.5% spore formation) and *Cbei*_*pde* (6.33% spore formation). Notably, low incidence of endospores was more pronounced in *Cbei*_*disA* than in *Cbei*_*pde*. Scanning electron microscopy further highlighted the abundance of bulging endospores in *Cbei*_p459 than in *Cbei*_*disA* and *Cbei*_*pde* at 24 h (Figure 7). As observed with phase contrast microscope, endospore formation was more prevalent in *Cbei*_*pde* than in *Cbei*_*disA*. Further, cell rupturing was observed in *Cbei*_*pde* but not in *Cbei*_*disA* and *Cbei*_p459 (Figure 7). Additionally, whereas the cells of *Cbei*_p459 appeared shorter, those of *Cbei*_*disA* and *Cbei*_*pde* were slender and longer, particularly *Cbei*_*disA*.

### *Cbei*_*disA*, *Cbei*_*pde*, and *Cbei*_p459 showed different responses to butanol and KCl

**FIGURE 8:**
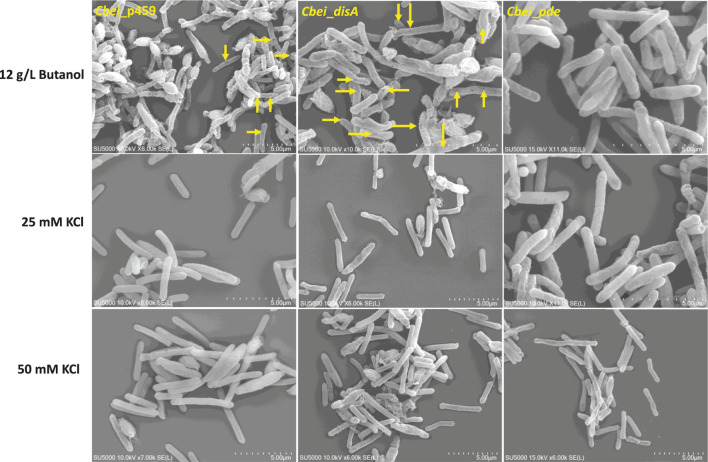
The effects of butanol (12 g/L) and KCl (25 and 50 mM) on *Cbei*_*disA*, *Cbei*_*pde,* and *Cbei*_p459. Arrows denote the presence of holes on cells. Striations were predominant in *Cbei*_*pde* treated with butanol and 25 mM KCl and *Cbei*_p459 treated with 25 and 50 mM KCl.

**FIGURE 9:**
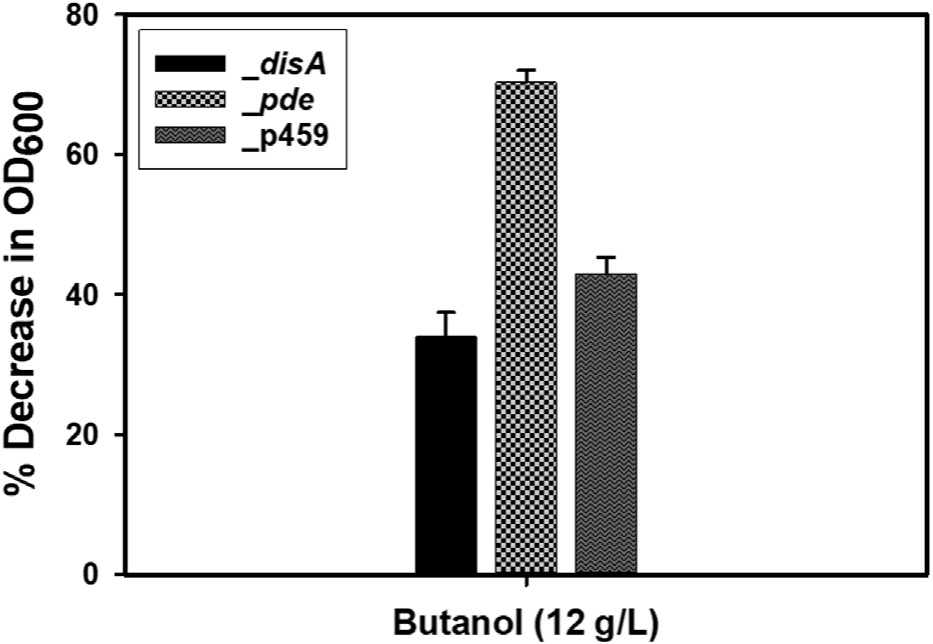
Percentage decreases in optical densities following butanol (12 g/L) challenge. Cloning and expression of *disA* in Cbei appeared to enhance tolerance to butanol. Data is presented as the mean of three biological replicates (n = 3). Error bars represent standard deviation.

Given the role of c-di-AMP in sensing cell membrane damage and the role of butanol in membrane damage (Commichau et al., 2015; Cray et al., 2015; Gardner and Grosser, 2024), we assessed the effect of butanol and membrane-damaging KCl concentrations on *Cbei*_*disA* and *Cbei*_*pde* compared to *Cbei*_p459. Shock addition of 12 g/L butanol to the culture medium at 24 h exerted considerable stress on all three strains of *Cbei* studied (Figure 8). Electron microscopy revealed extensive rupturing in *Cbei*_p459 with pronounced appearance of cell debris around endospores. Whereas severe butanol-mediated damage was observed for *Cbei*_*disA* and *Cbei*_*pde*, endospores were considerably less abundant in both strains, which retained their characteristic longer and slender morphologies. The cells of *Cbei*_p459 and *Cbei*_*disA* bore tiny holes whereas those of *Cbei*_*pde* were characterized by pronounced striations/markings—likely due to shrinking—that are absent in cells grown without butanol supplementation (Figure 7). The appearance of holes on the cells of *Cbei*_p459 and *Cbei*_*disA* was more pronounced in the latter. The morphologies of KCl-challenged cells indicate that butanol and KCl both negatively exert damaging effects on the *Cbei* cell membrane. However, micrographs suggest that they cause these effects via different mechanisms. When challenged with 25 mM KCl, the presence of striations was pronounced on cells of *Cbei*_p459 and *Cbei*_*pde*. On the other hand, striations were less prominent on cells of *Cbei*_*disA*, which appeared smaller and flaccid with more pronounced rupturing than *Cbei*_p459 and *Cbei*_*pde* (Figure 8). When 50 mM KCl was added to the cultures, cell rupture was observed in the cultures of all three strains of *Cbei* studied, with *Cbei*_p459 exhibiting a greater preponderance of striations (Figure 8). Apparently, cell rupture was more pronounced in *Cbei*_*disA*. Interestingly, both *Cbei*_*disA* and *Cbei*_*pde* had smaller cells than *Cbei*_p459 with 50 mM KCl. Flat and flaccid cells were more abundant in cultures of *Cbei*_*pde* challenged with 50 mM KCl than those of the same strain challenged with 25 mM KCl and 12 g/L butanol (Figure 8), and the unchallenged cells (Figure 7). Additionally, 8 hours after butanol (12 g/L) challenge, the optical densities of *Cbei*_*disA*, *Cbei*_p459, and *Cbei*_*pde* reduced 34%, 43%, 70%, respectively (Figure 9).

### Protein sequence analysis

Inhibition of solvent biosynthesis enzymes by exogenously supplemented c-di-AMP *in vitro* warranted a search of the *Cbei* proteome for c-di-AMP binding proteins. The results revealed 120 candidate proteins that harbor conserved c-di-AMP binding motifs (Supplementary Table S2). Among these, 35 proteins (29.2%) are involved in central metabolism and solventogenesis. Notably, three alcohol dehydrogenases including BdhA (Cbei_2421) with established involvement in butanol biosynthesis, hydrogenase (HypE), and [FeFe] hydrogenase, group A (Cbei_3796), which are known to take part in the ABE pathway (Tracy et al., 2012; Zhao et al., 2020) were found to contain c-di-AMP binding motifs (Tables 2; Supplementary Table S2). Additionally, 15—i.e., 42.9%—of the proteins with c-di-AMP binding motifs involved in central metabolism are either associated with glycolysis or are directly involved in the ABE pathway (Tables 2; Supplementary Table S2). As expected, 34 (28.33%) of the proteins bearing c-di-AMP binding motifs are involved in transport while 14 (11.7%), 12 (10%), 9 (7.5%), 8 (6.7%), 5 (4.2%), and 3 (2.5%) are involved in cell wall synthesis/cell membrane biogenesis, cell motility and signal transduction, sporulation and stress response, replication andtranslation, and c-di-AMP synthesis and degradation, respectively.

**TABLE 2:** Select proteins with c-di-AMP binding motifs in *Cbei*.

Protein	Pathway/Function	Protein ID
Iron-containing alcohol dehydrogenase (BdhA)	Butanol biosynthesis	WP_012058168.1
Iron-containing alcohol dehydrogenase	Putative butanol biosynthesis	WP_012060249.1
Iron-containing alcohol dehydrogenase	Putative butanol biosynthesis	WP_012058163.1
Aldehyde dehydrogenase	Aldehyde/alcohol metabolism	WP_012059995.1
Aldehyde dehydrogenase	Aldehyde/alcohol metabolism	WP_012060202.1
Hydrogenase formation protein (HypE)	ABE pathway	WP_012059198.1
[FeFe] hydrogenase	ABE pathway	WP_012059961.1
Phosphoenolpyruvate synthase	Glycolysis	WP_012058290.1
Pyruvate kinase	Glycolysis	WP_011968744.1
NADP-dependent GAPDH	Glycolysis	WP_012058783.1
Stage 0 sporulation family protein	Sporulation	WP_011967480.1
Stage V sporulation protein D	Sporulation	ABR33752.1
DHH family cyclic di-AMP phosphodiesterase	C-di-AMP degradation	WP_012061231.1
CdaA; c-di-AMP synthetase	C-di-AMP biosynthesis	ABR32390.1
ABC transporter permease	Transport	WP_012060301.1
Major facilitator symport transporter	Transport	WP_012060076.1

GAPDH, glyceraldehyde-3-phosphate dehydrogenase.

## Discussion

Having previously shown that knockdown of *disA* increased butanol production and delayed sporulation in *Cbei* (Ujor et al., 2021), our aim in this study was to determine if and to what extent c-di-AMP—the product of DisA—directly affects butanol production in this organism. Plasmid-borne expression of c-di-AMP-producing DisA and c-di-AMP-hydrolyzing Pde was used to assess the effect of intracellular c-di-AMP levels on butanol production in *Cbei*. Further, we assayed for the activities of enzymes central to solvent biosynthesis with and without c-di-AMP supplementation. Additionally, we measured the expression levels of genes relevant to butanol biosynthesis, sporulation, signal transduction, and central metabolism. Our results show that both high and low physiological levels of c-di-AMP severely impair butanol and acetone biosynthesis in *Cbei*.

This effect was particularly pronounced in the c-di-AMP-replete background (*Cbei*_*disA*), where biosynthesis of both acetone and butanol were almost completely inhibited. Accordingly, high and low levels of c-di-AMP impaired sporulation, with high c-di-AMP levels exerting a far more severe inhibitory effect on sporulation. The results are discussed under different subheadings for clarity.

### Potential link between c-di-AMP levels and sporulation in *Cbei*

Spo0A, the master regulator of sporulation in solventogenic clostridia exerts a strong positive effect on butanol biosynthesis (Long et al., 1984; Diallo et al., 2021). Both *Cbei_disA* and *Cbei_pde* exhibited poor sporulation relative to *Cbei*_p459. Oppenheimer-Shaanan et al. (2011) showed that reduced intracellular c-di-AMP levels in *B. subtilis* delays sporulation. While this is in agreement with the delayed sporulation observed for *Cbei_pde*, the more severe delayed sporulation observed for *Cbei_disA* suggests that dysregulated production of c-di-AMP—and not just reduction in c-di-AMP levels—blunts the rate of sporulation in *Cbei*. Hence, we infer that sub-optimal intracellular levels of c-di-AMP negatively affect sporulation, which may stall solventogenesis, particularly, butanol and acetone production in *Cbei*. The mRNA levels of *spo0A* in *Cbei*_*disA* and *Cbei*_*pde*, coupled with the reduced rate of spore formation in both strains suggests that overproduction of c-di-AMP does not impair *spo0A* transcriptionally (Table 1). Therefore, it is plausible that c-di-AMP likely affects Spo0A and indeed, sporulation posttranslationally.

Whereas Spo0A was not found to contain a known c-di-AMP binding motif, a Spo0A-related protein (WP_011967480.1; Tables 2; Supplementary Table S2) contains a c-di-AMP binding motif. It is plausible that c-di-AMP might affect other proteins involved in sporulation downstream of Spo0A. In fact, asides the Spo0A-related protein that contains a c-di-AMP binding motif, seven other proteins involved in sporulation (Supplementary Table S2) also contain c-di-AMP binding motifs. Thus, cloning and purifying Spo0A (Cbei_1712) and the Spo0A-related protein (WP_011967480.1) and assaying for c-di-AMP binding, in combination with a global protein pulldown study will shed more light on how c-di-AMP limits sporulation in *Cbei*_*disA* and Cbei_*pde*. More importantly, this promises to help delineate any interplays that exist at the nexus between c-di-AMP and Spo0A-mediated regulation of sporulation and solventogenesis in *Cbei*.

### Dysregulated production and hydrolysis of c-di-AMP impairs butanol and acetone biosynthesis and enhances ethanol production in *Cbei*

Careful examination of the butanol and acetone profiles show that *Cbei*_*pde* exhibited normal increases in butanol and acetone concentrations in the first 12 h of fermentation (Figures 3, 4). In fact, butanol and acetone concentrations in the cultures of *Cbei*_*pde* mirrored those of *Cbei*_p459 within the same period. Since both *disA* and *pde* were expressed under the *adc* promoter, which is auto-induced around 12 h when the culture pH drops following acid accumulation (i.e., acidogenesis; Tracy et al., 2012), it is logical therefore, to ascribe the drop-off in butanol and acetone production in *Cbei*_*pde* to the expression of *pde*. Notably, the vast majority of genes involved in solvent biosynthesis were significantly downregulated in *Cbei*_*pde* at both time points studied (24 and 36 h), albeit more at 36 h (Table 1; Supplementary Figures S1, S2).

In addition to the sharp drop in solvent production in *Cbei*_*pde*, the growth rate of this strain stopped mirroring that of *Cbei*_p459 after 12 h (Figure 2). Concomitantly, the mRNA abundance of pyruvate carboxylase gene (*pyc*; Cbei_4960), which codes for a key central metabolic enzyme, reduced drastically in *Cbei*_*pde* at 36 h (Table 1). Pyruvate carboxylase plays a crucial role in amino acid biosynthesis and lipid metabolism (Jitrapakdee and Wallace, 1999; Schär et al., 2010) and has been shown to be regulated by c-di-AMP in *B. subtilis*, *Listeria monocytogenes* and *Lactococus lactis* (Whiteley et al., 2017; Choi et al., 2017; Krüger et al., 2022). Taken together, these results are indicative of a gradual shift in gene expression profile, which leads to reduced growth and butanol and acetone biosynthesis in *Cbei*_*pde*. Given the membrane-damaging property of butanol and the cell rupture observed for *Cbei*_*pde* (Figure 7), we speculate that butanol biosynthesis and indeed, solventogenesis are aggressively scaled back in this strain, possibly to minimize the membrane damaging effect of butanol. Low intracellular levels of c-di-AMP lead to biosynthesis of inferior cell wall architecture, which leaves the cell membrane vulnerable to membrane damaging stressors (Wang et al., 2017) such as butanol.

Cell rupture observed for *Cbei*_*pde*, which produced at least 4.20-fold less butanol than *Cbei*_p459 supports this premise. More importantly, this might account for the arrest of solvent, particularly, butanol production in *Cbei*_*pde*. Given its chaotropic and ultimately, membrane damaging property (Figure 8), c-di-AMP being a sensor of membrane damaging stress may be recruited in *Cbei* to coordinate butanol biosynthesis. As such, impaired c-di-AMP accumulation in *Cbei*_*pde* and ultimately, the attendant inferior cell wall crosslinking (Wang et al., 2017) might elicit a regulatory sequence of cellular events that restrict butanol production.

Severe cell rupturing in *Cbei*_p459 relative to *Cbei*_*disA* following butanol challenge (Figure 8), lends weight to the notion that high intracellular levels of c-di-AMP in the latter may confer relatively higher tolerance to butanol. Whereas *Cbei*_*pde* did not exhibit as much cell rupturing as *Cbei*_p459 in butanol-supplemented cultures, electron microscopy suggests that this strain likely underwent severe shrinking (Figure 8). In fact, a significantly higher drop in cell density after butanol challenge of *Cbei*_*pde* (Figure 9) is indicative of a sharp drop in vegetative cell count. Comparatively, KCl caused greater cell shrinkage, whilst butanol brought about severe cell rupturing. Shrinking appears to be more severe in *Cbei*_*disA* than in *Cbei*_p459 and *Cbei*_*pde* treated with KCl. C-di-AMP binds to, and inhibits osmolyte uptake proteins (Cereija et al., 2021; Gardner and Grosser, 2024). This mechanism is adroitly deployed to control osmolyte uptake in response to the concentrations of osmotic stressors in the culture medium.

At the prevailing high intracellular c-di-AMP level in *Cbei*_*disA*, c-di-AMP would prevent potassium uptake in KCl-replete culture, which may account for greater shrinking of cells in 50 mM KCl-treated cultures of *Cbei*_*disA*. Similarly, with a likely weaker cell wall architecture, *Cbei*_*pde* appeared to undergo greater KCl-induced cellular damage than *Cbei*_p459 treated with 50 mM KCl (Figure 8). Despite a similar morphology between *Cbei*_*disA* and *Cbei*_*pde*, *Cbei*_*disA* exhibited more severe delayed sporulation than *Cbei*_*pde* and did not undergo rupturing in standard medium without an exogenous stressor. Resistance to cell membrane-damaging daptomycin has been linked to increased intracellular c-di-AMP concentration due to enhanced crosslinking of the cell wall (Wang et al., 2017; Zarrella and Bai, 2020). This phenomenon may account for the greater sturdiness observed for *Cbei*_*disA* when compared to *Cbei*_*pde*.

Compared to the control strain, the significantly reduced growth observed for *Cbei*_*disA* and *Cbei*_*pde*, irrespective of the sugar studied, is in concordance with previous reports that described c-di-AMP as “an essential poison” (Gundlach et al., 2015; Huynh and Woodward, 2016). Relative to other second messengers, c-di-AMP is distinctive in that, it is essential for growth in the bacteria that produce it, whilst being toxic at unusually high concentrations (Gundlach et al., 2015; Huynh and Woodward, 2016; Herzberg et al., 2023). This informed the choice to express *disA* and *pde* under the control of the auto-inducible *adc* promoter, which becomes active about 12 h of growth. This was intended to allow considerable cell mass to accumulate before the expression of both genes. Therefore, the considerably higher and lower c-di-AMP levels in *Cbei*_*disA* and *Cbei*_*pde*, respectively, most plausibly account for the reduced growth in both strains. While the lower solvent profiles observed for both strains may be ascribed to poor growth due to the negative effects of lower and higher physiological levels of c-di-AMP in *Cbei*_*disA* and *Cbei*_*pde*, this phenomenon does not sufficiently explain the drop in solvent production in *Cbei*_*pde* after 12 h, and the near inhibition of butanol and acetone biosynthesis in *Cbei*_*disA*.

Even with the diminished cell mass in cultures of *Cbei*_*disA*, this strain produced the same concentrations of ABE on glucose and arabinose as *Cbei*_*pde*. This is because, whilst butanol and acetone biosyntheses were severely diminished in *Cbei*_*disA*, ethanol production increased significantly, indicating selective improvement in ethanol biosynthesis at the expense of butanol and acetone biosyntheses. Butanol is the major NADPH disposal outlet in *Cbei* and other solventogenic clostridia for the purpose of redox balance (Tracy et al., 2012; Zhao et al., 2020; Agyeman-Duah et al., 2022). Thus, in the absence of butanol biosynthesis in *Cbei*_*disA*, the equally NADPH-dependent ethanol biosynthesis appears to be upregulated to address the ensuing potential redox imbalance. Lack of butanol (and acetone) biosynthesis in *Cbei*_*disA* hints at specific inhibition of both processes. Given the essentiality of c-di-AMP to bacteria that produce it (Gundlach et al., 2015; Huynh and Woodward, 2016; Herzberg et al., 2023), in addition to a likely response to cell rupturing in *Cbei*_*pde* (discussed earlier), marked and rapid reduction in physiological levels of c-di-AMP following *pde* expression might disrupt the progression of solventogenesis in this strain.

### C-di-AMP inhibits solventogenic enzymes of *Cbei in vitro*

The negative effect of c-di-AMP on the activities of ADC, BDH, BDDH, and to some extent CoAT, suggests that excess intracellular levels of c-di-AMP may counteract acetone and butanol biosynthesis at the protein level in *Cbei*. This result points to possible direct interaction between c-di-AMP and ADC, BDH, and BDDH. Intracellular concentrations of c-di-AMP in bacteria, which are estimated between 1 and 5 μM (Oppenheimer-Shaanan et al., 2011) are tightly controlled via adroit regulation of c-di-AMP-synthesizing diadenylate cyclases (such as DisA) and c-di-AMP-hydrolyzing phosphodiesterases (such as Pde). Remarkably, 5 nM c-di-AMP led to at least 55%, 45%, and 70% loss of ADC, BDH, and BDDH activities (Figure 5), respectively, whilst 50 nM c-di-AMP almost completely abolished the activity of each enzyme (ADC, BDH, and BDDH). Perhaps the physiological levels of c-di-AMP in *Cbei* are more tightly controlled with a significantly lower maximum threshold. More importantly, given the direct roles of ADC, BDH, and BDDH in acetone and butanol production, these results point to a possible role of c-di-AMP in regulating butanol and acetone biosynthesis in *Cbei*. This notion is supported by increased ethanol production in cultures of *Cbei*_*disA* (Figures 3, 4), given that ethanol is not as membrane damaging as butanol. Further, with the exception of coenzyme A transferase genes (which were upregulated), the genes for butanol biosynthesis enzymes were mostly downregulated in *Cbei*_*disA* at 24 h but were strongly upregulated in this strain at 36 h (Table 1). Further, *spo0A* and *adc* (involved in acetone production) were both significantly upregulated in *Cbei*_*disA* at 24 and 36 h. Nonetheless, these patterns (*i.e*., upregulated genes) did not amount to butanol or acetone production in this strain, thus, pointing to a possible underlying posttranscriptional c-di-AMP-mediated effect as the underpinning for this trend (*i.e*., lack of butanol and acetone production despite relevant gene upregulation). Increased ethanol production in *Cbei*_*disA* relative to *Cbei*_p459 and *Cbei*_*pde* suggests that an unidentified alcohol dehydrogenase that may not be affected by c-di-AMP, and with greater affinity for acetaldehyde as substrate than butyraldehyde is likely upregulated in *Cbei*_*disA* to dispose of excess NAD(P)H in the absence of butanol production.

Given the established ability of c-di-AMP to bind and inhibit a wide range of proteins (Cereija et al., 2021; Gardner and Grosser, 2024), coupled with the observed inhibition of ADC, BDH, and BDDH *in vitro*, c-di-AMP may directly affect solventogenic enzymes in *Cbei* at abnormally high intracellular concentrations. A search of the *Cbei* proteome revealed that BdhA, which participates in the terminal step of butanol biosynthesis (Tracy et al., 2012; Zhao et al., 2020; Agyeman-Duah et al., 2022) contains a c-di-AMP binding motif (Table 1; Supplementary Table S2). Interestingly, among the genes studied, *bdhA* was the most upregulated butanol dehydrogenase gene in *Cbei*_*disA* at 36 h (Table 1), when a strong upregulation was observed for most of the solventogenic genes studied. Although this is no confirmation that BdhA is the predominantly active butanol dehydrogenase at 36 h in *Cbei*_*disA*, the expression level and the presence of c-di-AMP binding motif in BdhA support the likelihood that excess intracellular c-di-AMP may disrupt some proteins involved in solventogenesis. Notably, seven other proteins involved or putatively involved in the ABE pathway were found to contain c-di-AMP binding motifs, thus, indicating that c-di-AMP may likely participate in the regulation of solventogenesis in *Cbei*.

It is important to note that the activities of some solventogenic enzymes (e.g., ADC and CoAT) in which c-di-AMP binding motifs were not found were also inhibited *in vitro* by c-di-AMP. It is either that c-di-AMP binds to motifs in these proteins that are not yet defined, or, that the activities of these enzymes are disrupted by c-di-AMP via a different mechanism. For example, the c-di-AMP-binding RCK_N domain of *B. subtilis* KtrA has been shown to also bind to ATP, ADP, NAD^+^, and NADH (Corrigan et al., 2013). Among the solventogenic enzymes studied, BDH and BDDH are NAD(P)H-dependent, which might be the basis for c-di-AMP-mediated inhibition of enzyme activities in the respective assays. However, this does not explain the basis for c-di-AMP mediated inhibition of ADC and to a lesser degree, CoAT. Cloning and purifying these enzymes and repeating these assays with c-di-AMP supplementation will provide additional insights as to how c-di-AMP inhibits the respective activities. More importantly, crystallographic analysis and substrate binding assays with purified enzymes in the presence of c-di-AMP will establish c-di-AMP binding as well as identify the potential binding pockets and motifs. Eight proteins central to the glycolytic pathway in *Cbei* were found to contain c-di-AMP binding motifs (Table 2 and Supplementary Table S2). Potential inhibition of some of these proteins will likely impair growth (and ultimately, solventogenesis), which may explain the particularly poor growth observed for *Cbei*_*disA*. We are currently deploying a global protein pull-down approach to identify the broader spectrum of c-di-AMP binding proteins in *Cbei*. This will reveal to what extent c-di-AMP might contribute to the regulation of solventogenesis in this organism.

### Conclusion

In conclusion, due to its capacity to detect membrane-damaging stress and the membrane damaging property of butanol, c-di-AMP may take part in the regulation of solventogenesis in *Cbei*. The results also suggest that c-di-AMP may coordinate the interplay between sporulation and butanol biosynthesis in *Cbei*. Demonstrating direct interaction between purified BDH, BDDH, and ADC and c-di-AMP will shed more light on how this second messenger inhibits butanol and acetone production in *Cbei_disA*. Furthermore, establishing direct interaction between c-di-AMP and Spo0A or other sporulation-related proteins, will prove instructive towards understanding the role of c-di-AMP in regulating sporulation and how this affects solvent biosynthesis in *Cbei*. Protein pull-down assay to identify and characterize c-di-AMP-binding proteins in *Cbei* will improve our understanding of the link between c-di-AMP-mediated regulation of solventogenesis and sporulation in *Cbei*.

## Data Availability

The original contributions presented in the study are included in the article/Supplementary Material, further inquiries can be directed to the corresponding author.
