# Supplementary material for: Dysregulated biosynthesis and hydrolysis of cyclic-di-adenosine monophosphate impedes sporulation and butanol and acetone production in Clostridium beijerinckii NCIMB 8052
Source: Front Bioeng Biotechnol. 2025 Feb 28;13:1547226. doi: 10.3389/fbioe.2025.1547226 (PMC11906698; doi:10.3389/fbioe.2025.1547226)
Supplement: Supplementary file 1 [file DataSheet1.pdf]

Dysregulated biosynthesis and hydrolysis of cyclic-di-adenosine monophosphate impedes butanol production in *Clostridium beijerinckii* NCIMB 8052

Marian M. Awaga-Cromwell, Santosh Kumar, Hieu M. Truong, Eric Agyeman-Duah, Christopher C. Okonkwo, Victor C. Ujor

Table S1: Primer Sequences used

Primer name	Primer sequence 5' → 3'	Gene function/name
**Cloning primers**		
Cbei_0127_Fv1	ATGAGAATAGAAAAGGGAATAGG	DNA integrity scanning protein A (*disA*; Cbei_0127)
Cbei_0127_Rv1	TCATATTTCCTTTTTTAATGAAAC	
Cbei_0127_Fv2	ATTAGGGCCCAGGAGGTTCAAGTTCATGGAATAGA AAAGGGAATAG	
Cbei_0127_Rv2	CGGCGGCGGCTCGAGTCATATTTCCTTTTTTAATGAAAC	
Cbei_5082_Fv1	GGCGGCGGCGGATGAATTGGATAACT ATGC	Phosphodiesterase (*pde*; Chei_5082)
Cbei_5082_Rv1	GGCGGCGGCGGTTATTCACCTATC CTTAAG	
Cbei_5082_Fv2	ATAAGGGCCCAGGAGGGGCGGCGG CATGAATTGGATAACTAT GC	
Cbei_5082_Rv2	GGCGCCGCGCCCTCGAGTTATTCACC TATCCTTAAG	
**qRT-PCR primers**		
Cbei_1712F	ATCAGCAGTAGGCCAAGATAAG	Asexual sporulation (*spo0A)*
Cbei_1712R	GTTTAATAGCTCTGCAGCAGTTC	
Cbei_1120F	GACTGGGATGGAAATGAGCTT	DNA-binding transcription factor activity (*sigE)*
Cbei_1120R	AGATAAGCAGCTATTAGTTATGGCA	
Cbei_4990F	TTGATGTACCTATACAGGAGGATAATG	DNA-binding transcription factor activity
Cbei_4990R	GCATCCTCTAACTCTTCCCTTAAT	
Cbei_0017F	TCGGTAAGGGATGAAGGAATTG	Histidine kinase KdpD
Cbei_0017R	CTATCCCACTTCCCGAATTCTC	
Cbei_3078F	CAGGTTTCCCTCCTTGTCTATTC	Histidine kinase
Cbei_3078R	TGATATGAGACGGCTAATTCCAA	
Cbei_0127F	CATAGAACGGCGCATAGAGTAG	DNA integrity scanning diadenylate cyclase (*disA)*
Cbei_0127R	GCTTGATTTGCCTTTCCAAGAA	
Cbei_5082F	GGATCTGCTCCCAAGGATTT	DHH family phosphoesterase (*pde)*
Cbei_5082R	GAAGGTTTGCTTGCAGGTATTT	
Cbei_0200F	GGGAAGAAGTGCTTTCGATGA	Adenylate cyclase activity (*cdaA)*
Cbei_0200R	CCTGTACTTTGCTCCATTACAATTAG	

Cbei_1538F	ATGCGTAAATTCTGGGAAAGT	DHH family phosphoesterase (*gdpP)*
Cbei_1538R	CGCCAAAGCTAATCTCTCTATTG	
Cbei_0305F	GACCTGTGGAGTTATAGAGAGAGA	Alcohol dehydrogenase/acetaldehyde dehydrogenase (*adhe1)*
Cbei_0305R	ATGGCTGTTGATGTTGGATTTG	
Cbei_4053F	GCAAGTCCGTGTGGAATATGA	Iron-containing alcohol dehydrogenase (*adhe2)*
Cbei_4053R	GAAGCATCTAATTTGGCAGGTATG	
Cbei_2421F	CGAAGGAGATCCATGGGATTTAG	NADH-dependent butanol dehydrogenase A (*bdhA)*
Cbei_2421R	CATGTGGATCCATCTCAGAACC	
Cbei_2181F	GCTCATAAGGTTGGTGCTGTA	NADPH-dependent butanol dehydrogenase (*bdhB)*
Cbei_2181R	GGCTCTCGCAATATCTCCATATC	
Cbei_1722F	ACAGAAGCAGGAATGGAAGTAG	NADPH-dependent butanol dehydrogenase (*bdhB)*
Cbei_1722R	TATCCAATCAGGCTCGAAGTTT	
Cbei_3835F	GCTTGGCTATCCAAAGCTATTT	acetoacetate decarboxylase (*adc)*
Cbei_3835R	CTAGAGGCTCGTGCTTATATCC	
Cbei_2654F	ATCAGCTCAACCAGGAGAAAC	CoA-transferase subunit A (*ctfA)*
Cbei_2654R	GCTTCAGGGCAAGTACCAATA	
Cbei_3833F	ACCTTCTATAGGGCTGCTACT	CoA-transferase subunit A (*ctfA)*
Cbei_3833R	CCAGGAGTCATTATGGCATCTC	
Cbei_2653F	AGGTGGATCTCCAATCACTTTAC	CoA-transferase subunit B (*ctfB)*
Cbei_2653R	ATCTACCTCTAAAGCGCCTAATG	
Cbei_3834F	GGGTGGCGCTATGGATTTA	CoA-transferase subunit B (*ctfB)*
Cbei_3834R	CCACTTGAGCCTTAGCAGTAA	
Cbei_4960F	GGTTCTAACACGAGTAGCCATTA	Pyruvate carboxylase
Cbei_4960R	TTAGATGCAGAAGGTCCAGAAG	

Table S2: Proteins with c-di-AMP binding motifs in *Cbei*

Protein	Protein ID	Gene locus
**Central metabolism and solventogenesis**		
**Iron-containing alcohol dehydrogenase**	**WP_012058168.1**	**Cbei_2421**
Aldehyde dehydrogenase family protein	WP_012059995.1	Cbei_3832
Iron-containing alcohol dehydrogenase family protein/ glycerol-1-phosphate dehydrogenase-like	WP_012060249.1	Cbei_4092
Pyruvate kinase	WP_011968744.1	Cbei_1412
NAD(P)H-dependent oxidoreductase	WP_012060847.1	Cbei_4693
NAD(P)/FAD-dependent oxidoreductase	WP_011967723.1	Cbei_0374
NAD(P)H-dependent oxidoreductase	WP_011967695.1	Cbei_0346
NAD(P)H-dependent oxidoreductase	WP_012060847.1	Cbei_4693
Phosphoenolpyruvate synthase	WP_012058290.1	Cbei_2063
Glucose-6-phosphate isomerase	WP_011967690.1	Cbei_0341
Ribose-5-phosphate isomerase RpiA	WP_012058583.1	Cbei_2367
Ribose-phosphate pyrophosphokinase	WP_012058303.1	Cbei_2076
NAD(P)/FAD-dependent oxidoreductase	WP_012058937.1	Cbei_2731
Short-chain-enoyl-CoA hydratase	WP_011967671.1	Cbei_0321
Short-chain-enoyl-CoA hydratase	WP_012058263.1	Cbei_2034
Iron-containing alcohol dehydrogenase	WP_012058163.1	**Cbei_1932**
Aldehyde dehydrogenase family protein	WP_012060202.1	Cbei_4045
2-hydroxyacyl-CoA dehydratase	WP_012058619.1	Cbei_2405
5-methyltetrahydropteroyltriglutamate--homocysteine S-methyltransferase	WP_012058545.1	Cbei_2329
Bifunctional UDP-sugar hydrolase/5'-nucleotidase/ 2'-phosphodiesterase	WP_011967983.1	Cbei_0635
Formate dehydrogenase accessory sulfurtransferase FdhD	WP_012059963.1	Cbei_3798
Pantoate--beta-alanine ligase	WP_012058820.1	Cbei_2609
6-phospho-beta-glucosidase	WP_011968045.1	Cbei_0700
Hydroxylamine reductase	WP_011968766.1	Cbei_1434
Acyl-CoA dehydratase activase	WP_012059923.1	Cbei_3758
NADP-dependent glyceraldehyde-3-phosphate dehydrogenase	WP_012058783.1	Cbei_2572
NADP-dependent glyceraldehyde-3-phosphate dehydrogenase	WP_012058499.1	Cbei_2282
Aldehyde dehydrogenase family protein	WP_012059995.1	Cbei_3832
Hydrogenase expression/formation protein HypE	WP_012059198.1	Cbei_3006
Methionine synthase	WP_012059289.1	Cbei_3100
Nicotinate-nucleotide--dimethylbenzimidazole phosphoribosyltransferase	WP_011968596.1	Cbei_1262
[FeFe] hydrogenase	WP_012059961.1	Cbei_3796
Xanthine dehydrogenase family protein molybdopterin-binding subunit	WP_012058215.1	Cbei_1984
[FeFe] hydrogenase, group A	WP_012060267.1	Cbei_4110
Asparagine synthase	WP_011968377.1	Cbei_1034
**Sporulation And Stress Response**		
Stage V sporulation protein D	ABR33752.1	Cbei_1578
PSP1 domain protein/Stage 0 sporulation family protein	WP_011967480.1	Cbei_0116
Spore photoproduct lyase	WP_012060518.1	Cbei_4361
Stage II sporulation protein D	WP_011967771.1	Cbei_0422
spore cortex biosynthesis protein YabQ	WP_011967458.1	Cbei_0094
Sensor histidine kinase YesM	WP_241402777.1	Cbei_2898

Sensor Histidine Kinase KdpD	WP_077836915.1	Cbei_2027
Sporulation protein YqfD	WP_011968184.1	Cbei_0841
**C-di-AMP synthesis and degradation**		
YbbR-like domain-containing protein; Cyclic di-AMP synthase regulator CdaR	WP_011967553.1	Cbei_0201
DHH family phosphoesterase/Cyclic di-AMP phosphodiesterase GdpP	WP_012061231.1	Cbei_5082
cdaA; c-di-AMP synthetase/ DisA_N domain protein	ABR32390.1	Cbei_0200
**Transport**		
ABC transporter permease	WP_012060301.1	Cbei_4144
ABC transporter permease	WP_012059308.1	Cbei_3121
ABC transporter substrate-binding protein	WP_012061191.1	Cbei_5042
Solute:sodium symporter family transporter	WP_011968716.1	Cbei_1383
Major facilitator symport transporter	WP_012061130.1	Cbei_4980
Sucrose-specific PTS transporter subunit IIBC	WP_012061161.1	Cbei_5012
Cation:dicarboxylate symporter family transporter	WP_012060501.1	Cbei_4344
Major facilitator symport transporter	WP_012060076.1	Cbei_3916
Major facilitator superfamily protein	WP_012060743.1	Cbei_4588
Major facilitator symport transporter	WP_012061207.1	Cbei_5058
ABC transporter ATP-binding protein	WP_012058998.1	Cbei_2793
Branched-chain amino acid ABC transporter permease	WP_011969088.1	Cbei_1764
Major facilitator symport transporter	WP_011968083.1	Cbei_0738
Branched-chain amino acid ABC transporter permease	WP_038458402.1	Cbei_2767
Cation diffusion facilitator family transporter/Divalent metal cation (Fe/Co/Zn/Cd) efflux pump	WP_012060930.1	Cbei_4777
Nucleobase:cation symporter-2 family protein	WP_012058640.1	Cbei_2426
Ammonium transporter	WP_011968214.1	Cbei_0871
Major facilitator symport transporter	WP_012060805.1	Cbei_4651
Amino acid ABC transporter permease	WP_012060328.1	Cbei_4171
ABC transporter permease subunit	WP_012059511.1	Cbei_3333
Efflux RND transporter permease subunit	WP_012059076.1	Cbei_2875
Cobalt ECF transporter T component CbiQ	WP_171787774.1	Cbei_3712
Amino acid permease	WP_012059456.1	Cbei_3276
Amino acid ABC transporter ATP-binding protein	WP_012058723.1	Cbei_2512
MATE family efflux transporter	WP_012058627.1	Cbei_2413
Magnesium transporter CorA family protein	WP_011967474.1	Cbei_0110
ClC family H^{+}/Cl^{-} exchange transporter	WP_012059132.1	Cbei_2937
Metal ABC transporter solute-binding protein, Zn/Mn family	WP_011967819.1	Cbei_0470
PTS sugar transporter subunit IIC	WP_012060837.1	Cbei_4683
Mannose/fructose/sorbose PTS transporter subunit IIB	WP_011969163.1	Cbei_1840
PTS sugar transporter subunit IIC	WP_011968808.1	Cbei_1478
PTS sugar transporter subunit IIC	WP_012060789.1	Cbei_4634
Sulfate/molybdate ABC transporter ATP-binding protein	WP_012058196.1	Cbei_1965
Na^{+}/H^{+} antiporter	WP_012058942.1	Cbei_2736
**Cell motility and Signal Transduction**		
TIR domain-containing protein	WP_012059010.1	Cbei_2805
Flagellar motor protein	WP_011968920.1	Cbei_1594
Flagellar motor protein	WP_012060430.1	Cbei_4273
Cadherin-like beta sandwich domain-containing protein	WP_012060850.1	Cbei_4696
Methyl-accepting chemotaxis protein	WP_012061115.1	Cbei_4965
Bifunctional diguanylate cyclase/phosphodiesterase	WP_011967999.1	Cbei_0651

Diguanylate cyclase	WP_012060855.1	Cbei_4701
Diguanylate cyclase	ABR35045.1	Cbei_2900
Osmosensitive K^+ channel signal transduction histidine kinase	ABR34196.1	Cbei_2027
**DNA replication and recombination**		
Recombinase family protein	WP_011968221.1	Cbei_0878
DNA-directed RNA polymerase subunit beta'	WP_011967507.1	Cbei_0145
DNA-directed RNA polymerase subunit beta	WP_011967506.1	Cbei_0144
YifB family Mg chelatase-like AAA ATPase	WP_011968521.1	Cbei_1184
Phosphoribosylformylglycinamidine synthase	WP_011968396.1	Cbei_1053
**DNA Transcription and Translation**		
Sigma-70 family RNA polymerase sigma factor	WP_012058461.1	Cbei_2237
Response regulator transcription factor	WP_012060746.1	Cbei_4591
Ribonuclease R	WP_038457150.1	Cbei_0633
YafY family protein; predicted DNA-binding transcriptional regulator YobV	WP_012059731.1	Cbei_3559
Sigma-54-dependent transcriptional regulator	WP_012061180.1	Cbei_5031
DhaR; sigma-54-dependent Fis family transcriptional regulator	WP_012058406.1	Cbei_2180
AraC family transcriptional regulator	WP_012060010.1	Cbei_3847
DEAD/DEAH box helicase	WP_011968315.1	Cbei_0972
MarR family winged helix-turn-helix transcriptional regulator	WP_012058318.1	Cbei_2092
Transcriptional regulator, RpiR family	ABR36914.1	Cbei_4807
DNA gyrase subunit A	WP_011967372.1	Cbei_0007
Cell wall metabolism sensor histidine kinase WalK	WP_011967884.1	Cbei_0535
Threonine--tRNA ligase	WP_011967643.1	Cbei_0841
**Cell wall synthesis/Cell membrane biogenesis**		
UDP-N-acetylmuramate dehydrogenase	WP_012061009.1	Cbei_4858
Glycosyltransferase	WP_012060886.1	Cbei_4732
Cyclopropane-fatty-acyl-phospholipid synthase family protein	WP_012060278.1	Cbei_4121
UDP-glucose/GDP-mannose dehydrogenase family protein	WP_012060892.1	Cbei_4738
Penicillin-binding transpeptidase domain-containing protein	WP_011967843.1	Cbei_0494
Glycerol-3-phosphate 1-O-acyltransferase PlsY	WP_012060002.1	Cbei_3839
N-acetylmuramoyl-L-alanine amidase	WP_012060852.1	Cbei_4698
UDP-glucose/GDP-mannose dehydrogenase family protein	WP_012060892.1	Cbei_4738
Murein biosynthesis integral membrane protein MurJ	WP_011968329.1	Cbei_0986
1-acyl-sn-glycerol-3-phosphate acyltransferase	WP_011969065.1	Cbei_1741
Dihydrodipicolinate synthase	ABR36883.1	Cbei_4776
Peptidoglycan-binding protein	WP_012059150.1	Cbei_2956

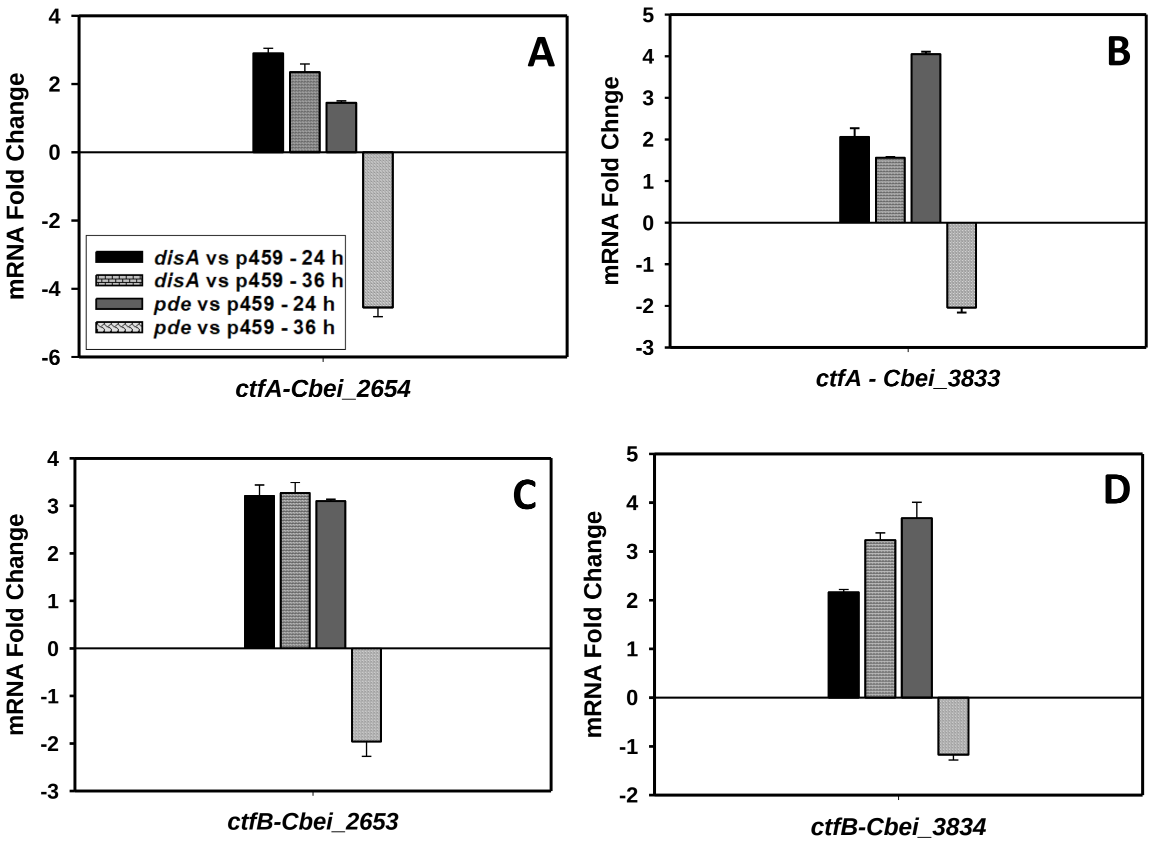

Fig. S1: Differential mRNA levels of coenzyme A transferase genes in *Cbei_pde* and *Cbei_disA* relative to *Cbei*_p459. A & B – *ctfA*; C & D – *ctfB*.

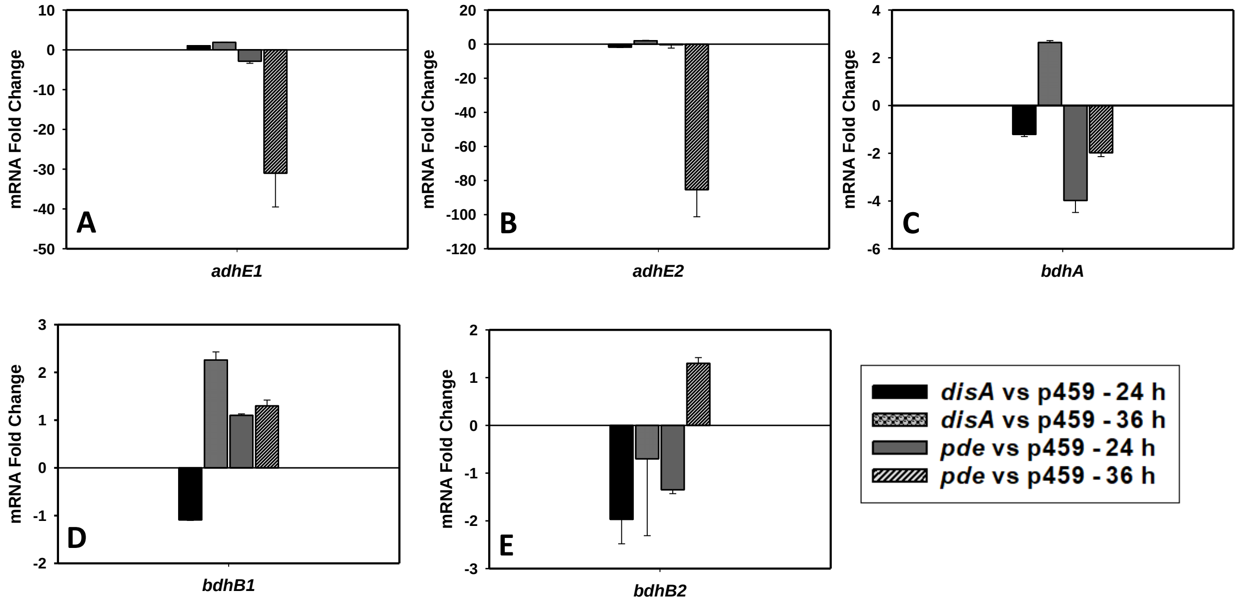

Fig. S2: Variations in mRNA levels of butanol dehydrogenase genes in *Cbei_pde* and *Cbei_disA* relative to *Cbei*_p459. A – *adhE1*, B – *adhE2*, C – *bdhA*, D – *bdhB1*, and E – *bdhB2*.
